# Fatigue resistance of monolithic and multilayer zirconia crowns using veneer layering or CAD-on technique

**DOI:** 10.1590/0103-6440202305687

**Published:** 2023-12-22

**Authors:** Alvim Gustavo Fasolin Tomm, Pablo Soares Machado, Lucas Saldanha da Rosa, Gabriel Kalil Rocha Pereira, Aloisio Oro Spazzin, Rodrigo Alessandretti

**Affiliations:** 1 Atitus Educação, Passo Fundo, Rio Grande do Sul, Brazil; 2 Post-Graduate Program in Oral Sciences, Center for Development of Advanced Materials, Division of Prosthodontics-Biomaterials, Federal University of Santa Maria (UFSM), Santa Maria, Brazil

**Keywords:** CAD-CAM, ceramic, multilayer structures, fractography, fatigue failure

## Abstract

This study aims to evaluate the fatigue resistance of monolithic zirconia (Yz) and multilayer ceramic structures using the CAD-on technique in different thicknesses. Fifty (N=50) standardized single crowns preparations were made in fiberglass-reinforced epoxy resin (NEMA grade G10), digitalized, and restorations were machined in CAD-CAM, composing 5 groups (n= 10): Control: 1.5 mm (milled zirconia framework + manual layered porcelain); Yz monolithic 1.5 mm; Yz monolithic 1.0 mm; CAD-on 1.5 mm; and CAD-on 1.0 mm (milled zirconia framework 0.5 mm thickness bonded by a low fuse ceramic to a milled lithium disilicate layer of 1.0 mm or 0.5 mm, respectively). The G10 bases were conditioned with 10% hydrofluoric acid; the crowns were air abraded with 110 μm alumina particles; and then luted onto each other with self-adhesive resin cement. A cyclic fatigue test was performed (initial load: 400N for 10,000 cycles, frequency of 20 Hz, step size of 200N) until failure, and the data was submitted to a survival statistical analysis. No failures were observed at Yz monolithic 1.5 mm. High and similar performance was observed for Cad-On groups and Yz monolithic 1.0 mm. The control group depicted the worst behavior. The Weibull modulus of CAD-on 1.5 mm was higher than the control while being similar to the other conditions. Both the monolithic systems and the CAD-on technique showed high and similar fatigue fracture behavior and survival rates, which were also higher than the control bilayer system. Both systems reduced the occurrence of delamination failures, making them suitable for clinical use.

## Resumo

Este estudo teve como objetivo avaliar o comportamento à fadiga de estruturas cerâmicas monolíticas de zircônia (Yz) e multicamadas utilizando a técnica CAD-on em diferentes espessuras. Cinquenta (N=50) preparos unitários padronizados foram confeccionados em resina epóxi reforçada com fibra de vidro (NEMA grau G10), digitalizados e as restaurações usinadas em CAD-CAM, compondo 5 grupos (n= 10): Controle: 1,5 mm (estrutura de zircônia fresada + porcelana estratificada manualmente); Yz monolítica 1,5 mm; Yz monolítica 1,0 mm; CAD-on em 1,5 mm; e CAD-on 1,0 mm (estrutura de zircônia fresada com 0,5 mm de espessura ligada por uma cerâmica de baixa fusão a uma camada de dissilicato de lítio fresado de 1,0 mm ou 0,5 mm, respectivamente). As bases do G10 foram condicionadas com ácido fluorídrico a 10%; as coroas foram jateadas com partículas de alumina de 110 μm; e então cimentadas uma sobre a outra com cimento resinoso autoadesivo. Foi realizado um teste de fadiga cíclica (carga inicial: 400N para 10.000 ciclos, frequência de 20 Hz, *step* de 200N) até a falha, e os dados foram submetidos a uma análise estatística de sobrevivência. Nenhuma falha foi observada para Yz monolítica de 1,5 mm. Desempenho alto e semelhante foi observado para os grupos Cad-On e Yz monolítica 1,0 mm. O grupo controle apresentou o pior comportamento. O módulo de Weibull do CAD-on 1,5 mm foi maior que o grupo controle, sendo semelhante às outras condições. Tanto os sistemas monolíticos quanto a técnica CAD-on apresentaram alto e semelhante desempenho mecânico e taxas de sobrevivência, que também foram superiores ao sistema bicamada de controle. Ambos os sistemas reduziram a ocorrência de falhas de delaminação, tornando-os adequados para uso clínico.

## Introduction

All ceramic restorations have become widely used for oral rehabilitation considering their biocompatibility, high esthetic appearance, and excellent mechanical behavior^1^. In this context, bilayer systems are still referred to as the gold standard for better mimicking natural teeth in the anterior region, combining the high mechanical strength of a ceramic in the framework, such as zirconia, and the esthetics of veneering porcelain. However, the difference between the properties of both ceramics (such as the elastic modulus and coefficient of thermal expansion) results in residual stress concentration at the interface, and consequent occurrence of chipping/delamination^2^, which are the most common failure types for such systems.

In order to reduce the occurrence of chipping, monolithic restorations produced via Computer-aided design-Computer aided manufacturing (CAD-CAM) emerged as an alternative treatment option^3^ ^,^ ^4^. Previous studies reported a low prevalence of failure rates for monolithic single crowns (less than 5%)^5^. Among the available materials for use in such a system, the first and second yttrium-stabilized zirconia (YSZ) generations are among the most used ceramics for monolithic crowns, since they present the highest mechanical strength when compared to other dental ceramics^6^. In addition, third-generation (4YSZ and 5YSZ) zirconia present an improved translucency, thus making it suitable for aesthetical restorations^7^. Moreover, lithium disilicate glass-ceramic is also a widely used option due to its versatility, which reunites excellent optical properties^8^ and satisfactory fracture strength^9^, even though it is not as high as YSZ^6^ ^,^ ^10^. 

New multilayer approaches have recently been proposed to reduce the differences between porcelain and zirconia, while still implementing a combination of materials to make restorations even more resistant to chipping when loaded. In this sense, the CAD-on system was introduced, in which the lithium disilicate glass-ceramic layer is also milled in the CAD-CAM system, and bonded to the zirconia framework using a fused ceramic which is simultaneously sintered with the lithium disilicate glass-ceramic. Some previous studies showed that the multilayer restorations produced with the CAD-on system presented comparable fracture strength to the monolithic zirconia system, as well as reduced chipping occurrence when compared to traditional bilayer restorations^11^ ^,^ ^12^. In addition, a previous clinical trial evaluated the efficacy of a CAD-on system after 5 years of follow-up and reported a 100% survival rate^13^. However, the available data about this system is still limited considering the fatigue behavior of this restorative set compared to monolithic and traditional bilayer restorations.

Finally, the mechanical and optical behavior of the restorations also depend on their thicknesses^8^ ^,^ ^14^ ^,^ ^15^. All-ceramic crowns enable a more conservative tooth preparation when compared to metal-ceramic crowns^16^. However, thinner ceramic thicknesses which are recommended by the manufacturers may affect the restoration's mechanical performance^14^, especially for multilayer approaches, in which each layer presents a very thin thickness. Thus, the evaluation of the minimal ceramic thickness that provides adequate fatigue behavior must also be considered.

Considering the above information, the objective of this study was to evaluate the fatigue failure load (FFL), number of cycles for failure (CFF), and survival probabilities of monolithic zirconia in different thicknesses and multilayer ceramic structures using a traditional veneer layer or the CAD-on technique in different thicknesses. The null hypotheses were that neither the^1^ restoration technique nor^2^ the restoration thickness would affect the fatigue behavior of the ceramic crowns.

## Material and methods

The ceramic materials used in the present in vitro study are described in Box 1. The experimental design is shown in Box 2. A simplified crown specimen geometry (N=50) was adopted for the mechanical test which was previously described by a previous study^17^. The sample size was based on a pilot study and defined as ten specimens per experimental group (n=10).

**Box 1:**
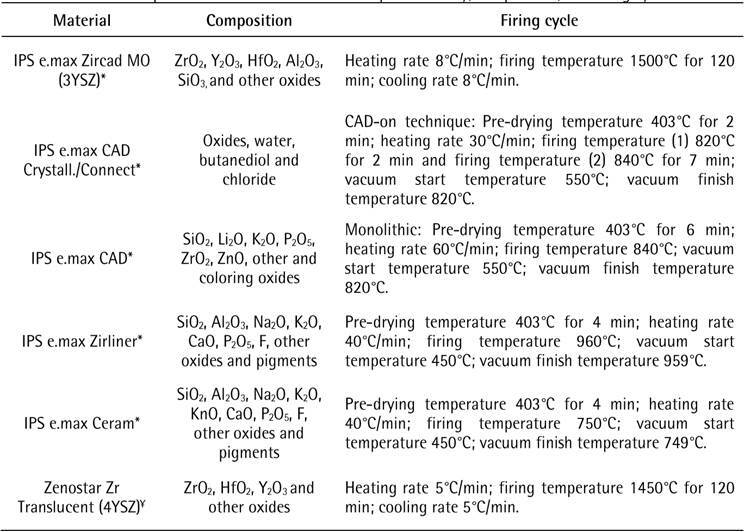
General description of the materials used in the present study, composition, and firing cycle

Information from manufacturers: *Ivoclar AG; ¥ Wieland Dental, Ivoclar AG.

**Box 2:**
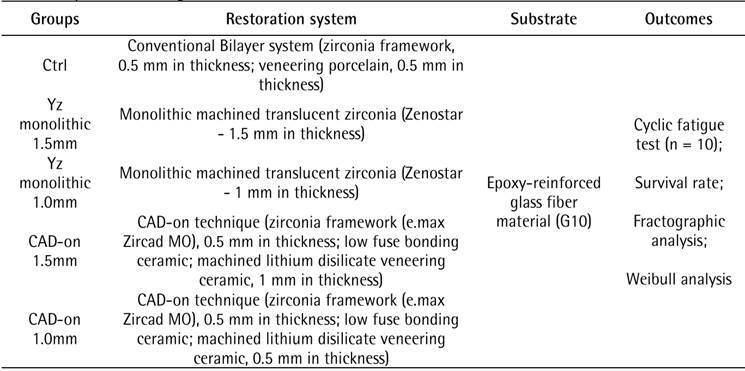
Experimental design

The preparations were milled using a fiberglass-reinforced epoxy resin (NEMA grade G10, Accurate Plastics Inc), validated by Kelly et al.^18^ as a dentin analog, in a precision mechanical lathe (Ergomat A25 - Vila Gea, São Paulo, Brazil), following the steps described by Schestatsky et al.^17^ The preparation’s geometry was simplified, presenting a conical shape, flat occlusal surface^19^, and axial walls at 8º of inclination with final space thickness of 1.5 mm for the restoration (occlusal, axial and chamfer). After that, all preparations were randomly assigned (random.org) into the groups considering the factors under study (Box 2).

Veneered zirconia crowns were first obtained as a control group (Ctrl) and were prepared by a single trained operator. Digital scanning (BioScan, BioArt, São Carlos, São Paulo, Brazil) of a reference G10 model was performed and the zirconia (IPS e.max ZirCAD MO, Ivoclar AG, Schann, Liechtenstein) framework (0.7 mm in thickness) was milled through a CAD-CAM system (INlab MC, Dentsply Sirona, Charlotte, USA) and then sintered according to the manufacturer's recommendations. Prior to the porcelain application, a thin layer (0.1 mm) of a low-fusing ceramic (IPS e.max Zirliner, Ivoclar AG) was applied over the framework to improve the bonding between the zirconia and porcelain. The bonding ceramic was fired according to the manufacturer’s instructions. The veneering ceramic (IPS e.max Ceram, Ivoclar AG) layer was applied by the stratification technique. The powder and build-liquid were mixed (1:1 ratio) and applied over the framework. An acetate matrix was used to help standardize the crown anatomy. The liquid excess was removed by an absorbent paper and ultrasonic vibration. Each applied layer was fired in a specific furnace until achieving the desired thickness for the group (0.7 mm for the veneer porcelain, 0.7 mm for the zirconia framework, 0.1 mm of low fusing ceramic; making 1.5 mm thickness in total).

Monolithic zirconia crowns (Zenostar Zr Translucent, Ivoclar AG) with two different thicknesses (1.0 mm or 1.5 mm) were also obtained by G10 model scanning and CAD-CAM milling processes. After finishing, the crowns were sintered in the Zirkonofen 600/V2 furnace (ZirkonZahn) according to the manufacturer's recommendations.

Next, two thicknesses were also considered for the CAD-on groups (1.0 mm and 1.5 mm). The zirconia framework (0.5 mm, IPS emax ZirCAD MO, Ivoclar AG) was obtained following similar procedures to the Ctrl group. The veneering lithium disilicate glass-ceramic (IPS e.max CAD, Ivoclar AG) was also milled by the CAD-CAM system according to each group's thickness (0.5 mm for the Cad-on 1.0 mm group; 1.0 mm for the Cad-on 1.5 mm group). A capsule of low fuse ceramic (IPS e.max CAD Crystall/Connect, Ivoclar AG) was mixed in a vibration device (Ivomix, Ivoclar AG) for 10 seconds, and then the material was applied on the lithium disilicate intaglio surface, which was immediately positioned over the zirconia framework. A load of 750 g was applied over the set and the excess of ceramic was removed with a microbrush. The lithium disilicate crystallization and firing of the low fuse ceramic were performed simultaneously in their specific furnace (Box 1).

All specimens were initially cleaned in an ultrasonic bath (1440 DA Odontobras, Ribeirão Preto, São Paulo, Brazil) for 5 minutes in distillate water and then air-dried. All crowns were tested over the respective preparation to evaluate the fit and dimensions. If any discrepancies were detected, the specimens would be replaced. The G10 preparations were conditioned with 10% hydrofluoric acid (IPS Ceramic etching gel, Ivoclar AG) for 20 s and then washed/air-dried for 30 s. The intaglio surface of the restorations was air-abraded with aluminum oxide particles (110 μm grain-size; Renfert do Brasil, Ribeirão Preto, São Paulo, Brazil) for 10 s, at 2 bar and 10 mm of distance.

The dual resin cement pastes (Multilink Speed, Ivoclar AG) were mixed (1:1 ratio) and applied to the crowns, which were then positioned over the G10 preparations. The resin cement excess was removed with a microbrush and a load of 750g was applied for 15 minutes. Finally, the bonded set was light-activated (Optilight Max, Gnatus Equipamentos Médico-Odontológicos Ltda, Ribeirão Preto, São Paulo, Brazil) for 60 s for each surface (occlusal, mesial, distal, buccal and lingual). The specimens were stored in distilled water for at least 24 hours before the mechanical tests.

**Figure 1:**
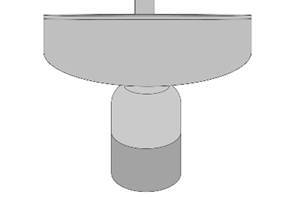
The illustrative figure of the fatigue test setup

Fatigue testing was executed with a step-stress methodology. An electric testing machine (ElectroPuls E3000; Instron Corporation, Norwood, USA) was used in which each crown (n= 10) was positioned centrally over a flat metallic base inside a cylinder matrix to ensure standardized positioning. The assemblies were submerged in water and a 40 mm diameter stainless-steel sphere applied the load at the center of the crowns. Adhesive tape (110 μm) was positioned between the loaded applicator and the crown to enhance the contact between them and avoid contact damage (Figure 1). Then, cyclical loads were applied at 20 Hz starting from 200 N for 5 000 cycles to adjust the testing assembly, followed by sequential increments with a step size of 200 N each 10 000 cycles until failure^17^ ^,^ ^20^. The specimens were detached from the base and submitted to light transillumination to search for cracks after each testing step. If cracks were detected, the specimens were considered as failed, and data regarding the fatigue failure load (FFL) and number of cycles for failure (CFF) were recorded. However, if cracks were not detected, the specimens were repositioned and the testing continued to a 2 800 N limit. If this limit was reached, the specimen was considered in the "survival" condition.

After failure, each crown was submitted to fractography analysis. First, a visual inspection with light transillumination of the region of failure was performed to categorize the failure pattern as 1- Hertzian cone-crack, when there is a circle format crack following the contact region between the load applicator and the crown surface; such pattern is indicative of origin at the occlusal surface^10^ ^,^ ^21^; 2- radial crack, where the crack propagates transversal to the area of tension generated by the load applicator/crown surface contact; such pattern is indicative of origin at the interface between materials (or between the zirconia framework and the porcelain veneer/lithium disilicate used at CAD-on technique; or between the monolithic restoration and the luting agent)^10^ ^,^ ^18^; 3- delamination, where there is detachment of the veneer material from the framework, suggestive of origin at the occlusal surface which than propagated to the interface between the veneer and the zirconia framework^2^ ^,^ ^22^. To prepare the specimens for the analysis and illustrate the fracture origin of radial cracks, the top part of the crown was cut horizontally 2 mm below the crown-G10 interface, thus including the occlusal and part of the axial wall of the restoration. After that, the radial crack was located via transillumination and marked with a pencil. To expand the radial crack until the complete fracture, the restorative fragment was loaded using a 3-point bending apparatus in a universal testing machine (EMIC DL 2000, São Jose dos Pinhais, Brazil), with an 8 mm span and a chisel-shaped load applicator (Load cell 1KN). The load was incrementally increased (crosshead speed of 0.5 mm/ min) until the complete propagation of the initial crack. Then, representative specimens were selected (n= 3) and submitted to scanning electron microscopy (SEM) analysis (Vega3; Tescan, Brno, Czech Republic) at 150× and 500× magnification.

FFL and CFF were submitted to survival analysis using the Kaplan-Meier and Mantel-Cox post hoc tests using a statistical software program (IBM SPSS Statistics, v21; IBM Corp, Armonk, USA) (α=.05). Survival rates were calculated for both parameters in the different testing steps. A Weibull analysis of this data was performed (Super-SMITH; Wes Fulton, Lake Arrowhead, USA) to assess the mechanical structural reliability of each evaluated ceramic by obtaining the Weibull moduli and its respective 95% confidence interval for both outcomes. Statistical differences for the Weibull moduli were obtained by using the maximum likelihood approach in which the overlap of confidence interval indicates statistical similarities and its absence points to statistical differences^23^. The fractographic pattern of failed crowns was descriptively analyzed.

## Results

The fatigue data were tested for parametric distribution and homoscedasticity using the Kolmogorov-Smirnov and Levene tests, respectively. The Kaplan-Meier (Mantel-cox post-hoc test) and Characteristic strength Weibull analysis (Table 1) showed that the different restorative assemblies had statistically significant differences in fatigue performance. The control condition had the worst fatigue performance (*P* <.001) for both outcomes (FFL and CFF), while the monolithic restorations with 1.5 mm thickness had the highest fatigue performance (100% survival). The CAD-on bilayer restorations with 1.5 mm thickness had the second-best performance (*P*<.001), followed by CAD-on bilayer restorations with 1.0 mm thickness (similar to CAD-on 1.5mm - *P* =.088) and monolithic restorations with 1.0 mm thickness (similar to both CAD-on groups - *P* =.173) (Table 1).

**Table 1:** Results from fatigue test depicting fatigue failure load (FFL) and cycles for failure (CFF)

Groups	FFL			CFF		
	Kaplan-Meier analysis and Mantel-cox posthoc tests*	Weibull analysis**		Kaplan-Meier analysis and Mantel-cox posthoc tests*	Weibull analysis**	
	Mean (Standard Deviation)	Characteristic Strength (95% Confidence interval)	Weibull modulus (95% Confidence interval)	Mean (Standard Deviation)	Characteristic Strength (95% Confidence interval)	Weibull modulus (95% Confidence interval)
Ctrl	1 400^C^ (298)	1 516^B^ (1 327 - 1 717)	5.68^B^ (3.26 - 8.90)	65 000^C^ (14 907)	70 720^B^ (61 271 - 80 836)	5.27^B^ (3.02 - 8.28)
Yz monolithic 1.5mm	All specimens of this condition survived the test, being excluded from the statistical analysis.					
Yz monolithic 1.0mm	2 340^B^ (327)	2 477^A^ (2 272 - 2 685)	8.77^AB^ (5.00 - 13.89)	112 000^B^ (16 363)	118 835^A^ (108 553 - 129 252)	8.40^AB^ (4.79 - 13.3)
CAD-on 1.5mm	2 680^A^ (253)	2 762^A^ (2 671 - 2 856)	22.41^A^ (11.74 - 37.97)	129 000^A^ (12 649)	133 108^A^ (128 548 - 137 807)	21.5^A^ (11.26 - 36.43)
CAD-on 1.0mm	2 560^AB^ (280)	2 671^A^ (2 524 - 2 816)	13.53^AB^ (7.46 - 22.02)	123 000^AB^ (13 984)	128 517^A^ (121 191 - 135 810)	12.99^AB^ (7.16 - 21.14)

* Different letters in these columns indicate statistical differences between evaluated conditions depicted by Kaplan-Meier and Mantel-Cox post-hoc tests (α = 0.05).

** Different letters in these columns indicate statistical differences between evaluated conditions depicted by Weibull Analysis, based on the absence of overlap of 95% confidence intervals (maximum likelihood estimation).

**Figure 2:**
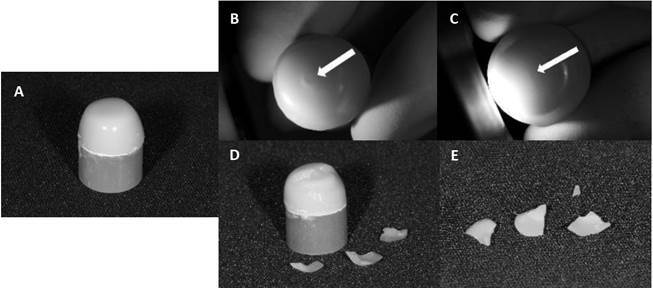
Representative figures of different failure patterns observed on crowns that failed during the fatigue test. a) specimen before testing. b) Hertzian cone-crack (indicated by the white arrow) which originates at the occlusal surface. c) radial crack (indicated by the arrow) with origin at interface between materials (between Yz framework and porcelain veneer/lithium disilicate used in CAD-on technique; or between monolithic restoration and luting agent). d) and e) delamination with origin at the occlusal surface which propagated to interface between a porcelain veneer and Yz framework

The Weibull modulus for both FFL and CFF data indicates superior statistical structural reliability for CAD-on 1.5 mm. The survival rates (Table 2, Figure 2) showed that all crowns of the control condition failed (0% survival rate) before any of the other evaluated conditions. At the test limit thresholds (2 800 N; 135 000 cycles), the crowns of the CAD-on 1.5 mm group had a survival probability of 70%, while the crowns of the CAD-on 1.0 mm and the monolithic restorations with 1.0 mm thickness groups had 30% and 10% survival probabilities, respectively (Table 2).

**Table 2:** Survival rates obtained in the Kaplan-Meier survival test, which indicates the probability of the specimens of such condition to exceed the respective fatigue failure load (FFL) and number of cycles for failure (CFF) step without failure, and its respective standard error values

Groups	FFL (N) / CFF (Count)													
	200/5 000	400/15 000	600/25 000	800/35 000	1 000/45 000	1 200/55 000	1 400/65 000	1 600/75 000	1 800/85 000	2 000/95 000	2 200/105 000	2 400/115 000	2 600/125 000	2 800/135 000
Ctrl	1	1	1	1	0.80 (0.13)	0.60 (0.16)	0.40 (0.16)	0.20 (0.13)	0.0	-	-	-	-	-
Yz monolithic 1.5mm	1	1	1	1	1	1	1	1	1	1	1	1	1	1
Yz monolithic 1.0mm	1	1	1	1	1	1	1	1	1	0.60 (0.16)	0.50 (0.16)	0.50 (0.16)	0.10 (0.10)	0.10 (0.10)
CAD-on 1.5mm	1	1	1	1	1	1	1	1	1	0.90 (0.10)	0.90 (0.10)	0.90 (0.10)	0.70 (0.15)	0.70 (0.15)
CAD-on 1.0mm	1	1	1	1	1	1	1	1	1	0.90 (0.10)	0.80 (0.13)	0.70 (0.15)	0.40 (0.16)	0.30 (0.15)

* The sign ‘-’ indicates the absence of a specimen of such condition being tested at this respective step.

**Figure 3:**
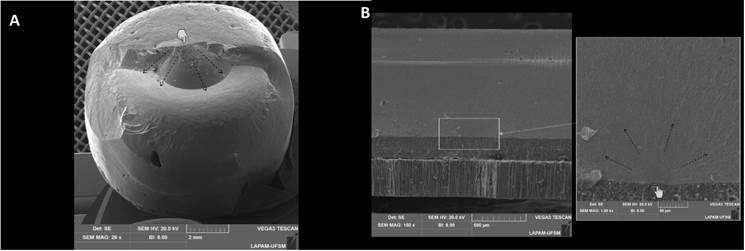
a) Representative SEM figures of specimens that failed by delamination, having failure originating in the occlusal portion of the crown, and being propagated between veneer ceramic and Yz framework. b) failure by radial crack, having failure originated in ceramic-epoxy resin interface and propagating to an occlusal portion of the crown. The pointer indicates failure origin, while arrows indicate the crack's propagation direction

The failure patterns (Table 3, Figure 2) showed that failures in the control condition mainly originated at the occlusal surface. Monolithic crown restorations with 1.5 mm thickness did not fail; meanwhile, crowns with 1.0 mm thickness presented all failures as radial cracks (100%). Bilayer restorations manufactured by the CAD-on technique showed some failures starting at the occlusal surface and most failures started at the interface as radial cracks. Fractographic analyses by SEM images are presented in Figure 3. Delamination failures occurred in bilayer crowns, with cracks originating in the occlusal portion, and propagating towards the porcelain until detachment from the zirconia framework. Some specimens also presented radial cracks with origin in the ceramic-G10 interface which propagated to the occlusal portion until the crown fracture.

**Table 3:** Results from the failure analysis illustrating the failure pattern observed in each condition and its occurrence prevalence in quantity and percentage.

Groups	Failure pattern			Survival at the end test
	Hertzian cone-crack	Radial crack	Delamination	
Ctrl	n = 6 (60%)	n = 2 (20%)	n = 2 (20%)	n = 0 (0%)
Yz monolithic 1.5mm	No failures (0%)	No failures (0%)	No failures (0%)	n = 10 (100%)
Yz monolithic 1.0mm	No failures (0%)	n = 9 (90%)	No failures (0%)	n = 1 (10%)
CAD-on 1.5mm	n = 1 (10%)	n = 2 (20%)	No failures (0%)	n = 7 (70%)
CAD-on 1.0mm	n = 4 (40%)	n = 3 (30%)	No failures (0%)	n = 3 (30%)

## Discussion

The fatigue tests showed that the monolithic zirconia and the CAD-on technique (1.0 and 1.5 mm thickness) presented higher fatigue fracture behavior than the conventional bilayer system. Thus, the first null hypothesis was rejected. This may be explained by the microstructure of each system. The veneering porcelain of the control group is known for its lower mechanical strength when compared to other glass and polycrystalline ceramics^6^. Also, when applied by the manual layering technique, porosities and defects are inherent along the microstructure. In addition, the interface of the conventional bilayer system is critical when considering mechanical performance. The bond strength between zirconia framework and veneering porcelain is also low due to the challenge of promoting good adhesion to polycrystalline materials^2^. In addition, the residual stresses at the interface due to thermal expansion coefficient differences between the ceramics also compromise the system, thereby increasing the risk of delamination^2^.

Monolithic zirconia crowns presented the highest fatigue fracture behavior in the present study when considering the 1.5 mm thickness (Table 3). The use of a monolithic system eliminates the difference of microstructures, and consequently its influence on mechanical properties present in multilayer restorations, thus reducing the chipping incidence and increasing the restoration's fracture strength^3^ ^,^ ^4^ ^,^ ^7^. In addition, the CAD-CAM milling process generates restorations with fewer internal defects when compared to the manual layering technique of veneering porcelain due to the nature of the milling blocks, which may also help to explain our findings. Furthermore, zirconia presents the highest mechanical strength among the available dental ceramics, even considering its third generation in which the translucency is increased^7^. Thus, the slow crack growth process was delayed for this system which presented high values for FFL, CFF, and even survival during the fatigue tests, mainly when the restoration thickness was increased (1.5 mm) (Table 1).

The CAD-on multilayer system also showed improved values fatigue failure load than the control group, even comparable to the monolithic restorations (Table 1). This may be explained by the use of a milled lithium disilicate glass ceramic as a veneering material which probably presented fewer defects in comparison to the manually layered porcelain due to its mechanized manufacturing by CAD-CAM^24^. Lithium disilicate glass-ceramic contains a 70% volume fraction of lithium disilicate crystals after firing^9^, being a versatile material that presents great esthetic and high mechanical performance^8^. The interface between the glass-ceramic and zirconia framework also seemed to be reinforced by the use of a fusion ceramic that probably filled the defects present^13^ ^,^ ^25^, which is corroborated by the less prevalent radial cracks for the CAD-on technique when compared to the conventional bilayer system (Table 3). It is important to note that no delamination was observed in the CAD-on group regardless of the crown thickness. Thus, the combination of zirconia/lithium disilicate glass-ceramic promoted high-performance restorations which may also be considered promising for clinical use.

The tested thicknesses also affected the fatigue behavior of the evaluated systems, so the second null hypothesis was also rejected. Monolithic zirconia restorations with 1.5 mm in thickness presented 100% survival at the end of the mechanical test, indicating superior performance when compared to 1.0 mm zirconia crowns (Tables 2 and 3). This is due to previous studies that indicated a positive influence of increased thickness on the fracture strength of dental ceramics^15^. However, it must be noticed that the monolithic zirconia and CAD-on multilayer crowns (1.0 mm in thickness) showed similar fatigue behavior under reduced thickness when compared to the 1.5 mm CAD-on group. Moreover, the thinner sets also showed high values for FFL and CFF and a considerable survival percentage at the end of the test (Table 3). Zimmermann et al.^15^ showed high fracture load values for ceramic and composite CAD-CAM materials when used with thicknesses of 1.0 mm and 1.5 mm, corroborating our findings. Hence, the use of such reduced thicknesses seems feasible for a clinical scenario, reducing the need for tooth preparation or in cases with limited interocclusal space.

As an in vitro study, it is necessary to point out some limitations. The use of simplified crowns did not evaluate concentrated tensions in cusps and fossa related to anatomic crowns. Thus, the failure pattern may have been affected by multidirectional loads which occur in a clinical scenario. Besides, different thicknesses were adopted for each multilayer system in the present study, however, it was necessary to follow the manufacturer instructions for conventional veneered zirconia and the CAD-on assemblies, which are used in the clinical scenario. It is also important to ponder that the higher number of specimens surviving the test on cad-on groups may have overestimated the structural mechanical reliability of such groups, which numerically depicted higher values for the Weibull modulus. Even so, there were no statistical differences in Weibull modulus between them and monolithic groups. Finally, the tested specimen geometry made it difficult to evaluate the radial crack origins (at the bottom of the framework or from the veneering material). However, such geometry was important to isolate the factors under study, thereby enabling to evaluation of only the system structure and thickness on the fatigue behavior of the sets. Thus, future studies considering other factors in a clinical scenario are encouraged.

## Conclusion

Both the monolithic systems and the CAD-on technique showed high and similar fatigue failure loads fracture behavior, which was also higher than the conventional bilayer system (zirconia framework + veneering porcelain). Both systems reduced the occurrence of delamination failures, making them suitable for clinical use.
